# MHC Genotyping by SSCP and Amplicon-Based NGS Approach in Chamois

**DOI:** 10.3390/ani10091694

**Published:** 2020-09-18

**Authors:** Sunčica Stipoljev, Elena Bužan, Barbora Rolečková, Laura Iacolina, Nikica Šprem

**Affiliations:** 1 Faculty of Agriculture, University of Zagreb, 10000 Zagreb, Croatia; sstipoljev@agr.hr (S.S.); lauraiacolina@gmail.com (L.I.); nsprem@agr.hr (N.Š.); 2 Faculty of Mathematics, Natural Sciences and Information Technologies, University of Primorska, 6000 Koper, Slovenia; 3 Environmental Protection College, 3320 Velenje, Slovenia; 4 Institute of Vertebrate Biology of the Czech Academy of Sciences, 60365 Brno, Czech Republic; roleckova@ivb.cz

**Keywords:** major histocompatibility complex, next-generation sequencing, Ion Torrent, *Rupicapra rupicapra*

## Abstract

**Simple Summary:**

The major histocompatibility complex is a family of genes of central importance in vertebrate adaptive immunity. Genes of the major histocompatibility complex are among the most polymorphic genes ever described in vertebrates, with their number differing greatly among species. Due to their functional significance and exceptional diversity, they represent excellent markers in evolutionary ecology and conservation. Traditional methods were commonly used for their genotyping, but the introduction of next-generation sequencing facilitated more accurate and reproducible genotyping of such polymorphic gene families. Nevertheless, due to the high polymorphism of the major histocompatibility complex genes, genotyping is inherently difficult. Here, we compared the performance of traditional and next-generation sequencing on genotyping of the major histocompatibility complex genes in chamois. Although the major histocompatibility complex system in chamois is quite simple, we found genotyping discrepancies between the two methods in 25% of individuals. Our results show that next-generation sequencing has a higher detection capacity and thus allows for more accurate genotyping of highly polymorphic genes.

**Abstract:**

Genes of the major histocompatibility complex (MHC) code for cell surface proteins essential for adaptive immunity. They show the most outstanding genetic diversity in vertebrates, which has been connected with various fitness traits and thus with the long-term persistence of populations. In this study, polymorphism of the MHC class II *DRB* locus was investigated in chamois with Single-Strand Conformation Polymorphism (SSCP)/Sanger genotyping and Ion Torrent S5 next-generation sequencing (NGS). From eight identified *DRB* variants in 28 individuals, five had already been described, and three were new, undescribed alleles. With conventional SSCP/Sanger sequencing, we were able to detect seven alleles, all of which were also detected with NGS. We found inconsistencies in the individual genotypes between the two methods, which were mainly caused by allelic dropout in the SSCP/Sanger method. Six out of 28 individuals were falsely classified as homozygous with SSCP/Sanger analysis. Overall, 25% of the individuals were identified as genotyping discrepancies between the two methods. Our results show that NGS technologies are better performing in sequencing highly variable regions such as the MHC, and they also have a higher detection capacity, thus allowing a more accurate description of the genetic composition, which is crucial for evolutionary and population genetic studies.

## 1. Introduction

In the conservation and management of species, genetic monitoring is essential to ensure that appropriate measures are taken to maintain the viability and adaptive potential of the population, particularly in the case of small and declining populations [1]. Although presumably neutral markers are often used to quantify genetic diversity, neutral variation is not enough to understand all mechanisms that shape the genetic variation of populations [2]. Studying the molecular polymorphism of adaptive genes (i.e., genes that directly influence fitness) can help to understand how adaptive genetic variation is generated and maintained within populations [3].

Among the most polymorphic genes in vertebrates are the major histocompatibility complex (MHC) genes, which are responsible for the adaptive immune response [4]. Polymorphism of the MHC genes has been shown to be related to the individual’s fitness and thus to the long-term persistence of populations, which makes these genes important markers for various fitness traits, including factors important for population viability, such as resistance to parasites, survival, and reproductive success [4]. The most pronounced polymorphism of the MHC molecules occurs in the amino acid residues that code for the antigen binding groove [5]. As a result of the function of MHC molecules, pathogen-driven balancing selection has generally been assumed as a major evolutionary force for maintaining MHC polymorphism [6], but it is also produced by gene duplication, resulting in extensive copy number variation [7]. Thus, the genetic diversity of MHC is not only characterized by extreme allelic polymorphism and high nucleotide diversity, but also by the number of duplicated genes.

The introduction of high-throughput sequencing (HTS) technologies, also known as next-generation sequencing (NGS), has allowed for a large-scale assessment of genetic variation at reasonable times and costs [8]. In addition, NGS methods improved our ability to genotype highly polymorphic multigene families such as the MHC [9,10,11].

Great effort has been devoted to MHC genotyping in evolutionary and population studies [12,13]. However, the task is still quite demanding and challenging due to the complex genomic organization and the high sequence variation of MHC loci, as well as the difficulties to separate true alleles from artefacts [14,15]. MHC genes, often in multiple copies, vary widely between and within species, making the identification of all alleles carried by an individual and the reconstruction of its multilocus genotype very challenging [16]. Consequently, the problems that cause significant difficulties in MHC genotyping are (i) the frequent gene duplications and variation between haplotypes in the number of loci within and between species, (ii) the difficult design of locus-specific primers, (iii) varying degrees of concerted evolution, and (iv) the presence of pseudogenes, which cause additional difficulties in identifying functional variants.

For MHC genotyping, different techniques such as Restriction Fragment Length Polymorphism, Single-Strand Conformation Polymorphism (SSCP), Denaturing Gradient Gel Electrophoresis, and Reference Strand-mediated Conformational Analysis in combination with cloning were used [17].

The SSCP method is based on the observation that single-stranded DNA fragments with different DNA sequences will assume sequence-specific conformation when electrophoresed under non-denaturing conditions [18]. Since a change in a single base is sufficient to cause changes in the tertiary structure of single-stranded DNA fragments, SSCP is able to detect single-base substitutions. The SSCP method has been proven robust, with a high sensitivity to detect DNA sequence variation for MHC genes [19,20], but it can encounter problems when the amplification of some alleles is less efficient or when certain alleles are difficult to distinguish [17]. It especially becomes problematic and unreliable if multiple co-amplifying copies are present in the sample [17].

Despite significant advances in HTS technologies, the genotyping of MHC systems and the ability to discriminate between true alleles and artefacts is more challenging as the number of co-amplifying genes increases with this method. Of the available HTS platforms, Ion Torrent and Illumina are among the most appropriate choices for MHC genotyping due to the ultra-high coverage and read lengths, offering the potential to overcome this limitation [16].

Here, we compared the performance of two methods, SSCP and Ion Torrent S5 sequencing, for genotyping highly polymorphic exon 2 of the MHC class II *DRB* gene in chamois, which encodes functionally important residues of the antigen-binding groove and therefore can be taken as a measure of functional diversity of *DRB* alleles [21].

## 2. Materials and Methods 

DNA from 28 chamois muscle samples was extracted with peqGOLD Tissue DNA Mini Kit (VWR International, Leuven, Belgium) according to the manufacturer’s protocol. All animals were legally harvested according to the Slovenian Hunting Law (Official Gazette of the Republic of Slovenia Ur. l. 16/04) and Croatian Hunting Law (Official Gazette of the Republic of Croatia 99/18) regular hunting allocations. Handling of animals and sampling were done according to the Ethical and Welfare Standards presented in the (Official Gazette of the Republic of Croatia 102/2017), Regulation on the Protection of Animals Used for Scientific Purposes (Official Gazette of the Republic of Croatia 55/13) and the Bioethical Committee for the Protection and Welfare of Animals of the University of Zagreb Faculty of Agriculture.

### 2.1. MHC Genotyping by SSCP/Sanger Sequencing

The Qiagen multiplex PCR kit was used to amplify the second exon of MHC class II *DRB* gene using primers HL030: 5′-ATCCTCTCTCTGCAGCACATTTCC-3′ and HL032: 5′-TCGCCGCTGCACAGTGAAACTCTC-3′ [22]. PCR was performed following the protocol described elsewhere [22,23] and verified by electrophoresis on ethidium bromide stained agarose gel. Successful PCR products were cloned according to the protocol by Bryja et al. [24] and, following an additional PCR step [22], they were evaluated by single-stranded conformational polymorphism analysis and sequenced by capillary electrophoresis in an ABI 3130 analyzer. Sequencing and allele identification were performed according to the protocol described in Čížková et al. [23]. The results were validated with GeneMapper v.5.0 (Thermo Fisher Scientific, Waltham, MA, USA) software. The total length of the 236 bp allele sequences was assembled using CodonCode Aligner software (version 1.6.3; CodonCode Corporation, Dedham, MA, USA) and aligned with ClustalW 4.0, implemented in MEGA 7 [25].

### 2.2. MHC Genotyping by An Amplicon-Based NGS Approach

Amplicon sequencing was performed in three runs using an Ion Torrent S5 system (Thermo Fisher Scientific). Amplification was carried out by using the same two primers HL030 and HL032. In order to identify individuals, forward primers were designed to include the following motifs: (i) Ion Torrent A adapter, (ii) unique IonXpress barcode, (iii) barcode “GAT” linker, and (iv) forward primer HL030. PCR amplification was performed in triplicates in 25 µL reaction mixtures containing a 5 µL DNA template, 5X reaction buffer, 5 µL Q solution (Qiagen, Hilden, Germany), 3 µM MgCl_2_, 0.25 mM dNTPs, 0.15 µM of each primer, and 0.08 U HotStarTaq (Qiagen). The PCR program included an initial denaturation step at 95 ℃ for 2 min, 40 denaturation cycles at 95 ℃ for 20 s, primer annealing at 60 ℃ for 30 s, and primer extension at 72 ℃ for 45 s, and a final elongation step at 72 ℃ for 10 min. PCR products from triplicates were pooled and purified with Agencourt AMPure XP beads (Agencourt Bioscience Corporation, Beverly, MA, USA). The concentrations of the pooled and purified amplicons were estimated with the Qubit 3.0 Fluorometer using the Qubit dsDNA High Sensitivity Assay Kit (Thermo Fisher Scientific). Then, amplicons were normalized to 5 ng, pooled and purified again with Agencourt AMPure XP beads. Size and quality of the pooled amplicons were verified using the Agilent DNA High Sensitivity Kit on the 2100 Bioanalyzer (Agilent, Santa Clara, CA, USA). The final library was normalized to 100 pM and sequenced with the Ion Torrent S5 on a 314 chip (Thermo Fisher Scientific).

Allele calling was conducted through the pipeline based on the Amplicon Sequence Assignment (AmpliSAS) web tool designed for high-throughput genotyping of duplicated polymorphic gene families, such as the MHC [26]. Initial quality and length filtering of raw data was performed with AmpliCLEAN by removing reads with a Phred quality score below 30 and all reads shorter than 279 bp and longer than 289 bp. AmpliSAS clusters true variants with their potential artefacts based on the platform-specific error rates. We used AmpliSAS default parameters for Ion Torrent sequencing technology: a substitution error rate of 0.5% and an indel error rate of 1%. Exact length was required for the dominant sequence within a cluster. From previous work on this species [22,27,28,29], we did not expect more than two *DRB* variants per individual, so we kept the “minimum dominant frequency” clustering threshold at default 25%. Variants with frequency lower than 1% within an amplicon were discarded.

The maximum number of reads per amplicon that can be processed by AmpliSAS is 5000; hence, the amplicons with more than 5000 reads were randomly sub-sampled.

The datasets generated and analyzed during the current study were deposited in the NCBI Sequence Read Archive (SRA, accession number PRJNA662939).

True *DRB* variants of the second exon were aligned and translated into protein sequences to check whether there was evidence for pseudogenes, such as the presence of premature stop codons. Three undescribed alleles discovered in this study (two of them validated by both methods) were named Ruru-DRB*41, Ruru-DRB*42, and Ruru-DRB*43 and deposited in GenBank with accession numbers MT813042–MT813044.

The evolutionary relationships between the alleles were analyzed by a median-joining network, as implemented in the software PopART [30]. The parameter ε was set to zero (default) to obtain a sparse spanning network. We compared frequencies of alleles obtained with the two genotyping approaches and estimated discrepancies in each individual’s genotypes between the two methods.

## 3. Results

The sequence of 236 bp (without primers) of the *DRB* exon 2 gene was obtained for all 28 chamois. After initial filtering of the raw Ion Torrent data, the amplicon coverage ranged from 656 to the maximum of 5000 reads allowed by AmpliSAS, with an average of 3191 ± 1996 (SD) reads. The coverage in our study was sufficient to separate true alleles from artefacts and obtain reliable genotypes. 

When clusters within an amplicon were ordered by descending per amplicon frequency (PAF), we observed a significant drop in frequency at the value of 3%, which probably represents the boundary between true alleles and artefacts. For one individual, we found chimeric variant at a frequency of 13%. Accordingly, we set the PAF threshold at 14% and removed the chimeric and low-frequency variants below this threshold (Figure S1 and Table S1).

Clustering sequencing errors with true variants increased the read depths of true variants and thereby allowed us to more easily distinguish alleles from low-frequency artefacts. On average, 54% of amplicon depths were assigned to alleles, and after clustering, this proportion increased to 83% (Figure S2). Ion Torrent tends to produce high rates of homopolymer indels, depending on the properties of the analyzed sequences. However, this had very little effect on the results of genotyping of chamois. The estimates of per amplicon variant frequencies after clustering were mainly affected by 1 bp substitution (Figure S3), which accounted, on average, for 21% of the coverage increase of true variants within the amplicon (Table S2). 

**Figure 1:**
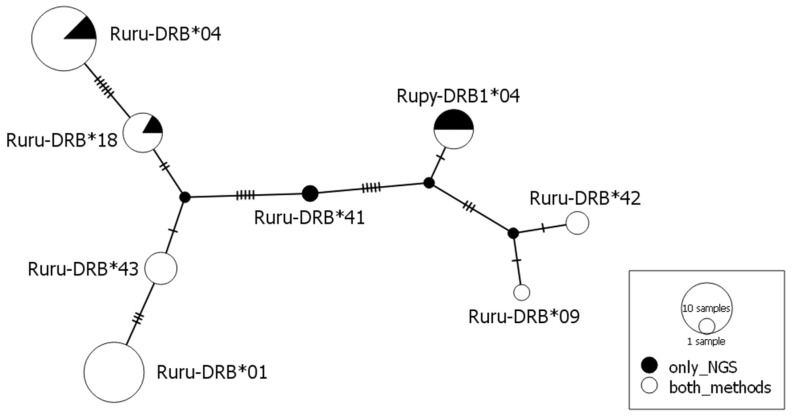
Median-joining network (ε = 0) of *DRB* alleles found in chamois. Alleles are represented by pie charts whose size is proportional to the number of individuals. The colors indicate the number of individuals that have particular alleles obtained with two genotyping methods. Number of mutations separating nodes is represented by slashes crossed with the network branches. Small black circles indicate hypothetical alleles, as predicted by the model.

**Figure 2:**
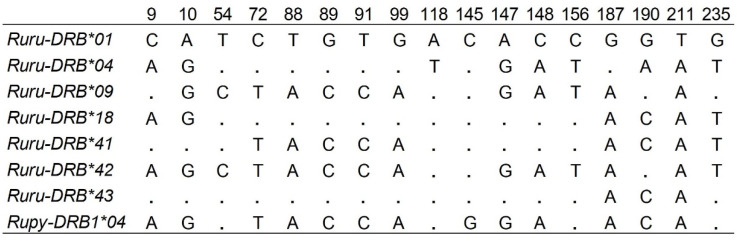
Alignment of the nucleotide sequences, only variable residues of the exon 2 of *DRB* alleles of chamois are shown. The codes before * represent the species abbreviation and gene name, and the numbers after * indicate allele numbers.

No stop codons were found in the nucleotide sequence of any allele identified by SSCP/Sanger sequencing and amplicon-based NGS, indicating that all alleles encoded for functional proteins. The allele identities were based on 17 (7%) variable nucleotide positions with the lowest number of mutations (two) between the alleles Ruru-DRB*09 and *42. The maximum number of 14 mutations was found between allele Ruru-DRB*01 and *42 (Figure 1 and Figure 2).

A total of eight alleles were identified in 28 individuals. Five of them had already been described [31,32], and three were new, not previously described alleles. We discovered seven alleles by SSCP/Sanger genotyping, and all of them were also detected by NGS sequencing. Further, NGS enabled detecting one more allele and extended the genotypes of six of the 28 individuals compared to SSCP/Sanger genotyping results (Table S3).

The maximum number of alleles per individual detected by both methods was two, which was consistent with the expectation of a single *DRB* locus from previous studies. A considerable variation in frequency between alleles was observed. For both the methods, the lowest frequencies were for Ruru-DRB*41 (0% and 2%, respectively) and Ruru-DRB*09 (2%), and the highest were for Ruru-DRB*01 (36% and 32%, respectively) (Table 1).

The number of called alleles per individual was consistent between the two genotyping methods in 75% of the cases. Discrepancies in the individual genotypes between the two methods were mainly caused by allelic dropout using the SSCP/Sanger analysis. Six out of 28 individuals (21%) were falsely classified as homozygous with SSCP/Sanger analysis due to dropout events. In addition to the allelic dropout, we had a false positive allele call for one individual with SSCP/Sanger analysis, which was due to a weak obtained sequence. In total, 25% of the individuals were identified as genotyping discrepancies. A summary of the *DRB* alleles found in each individual using both genotyping approaches is given in Table S3.

## 4. Discussion

Our results show that NGS technologies are better performing in the sequencing of highly variable regions such as the MHC and could offer real advantages compared to the SSCP/Sanger methodology. However, knowledge of the complexity of the MHC system in the study species and, accordingly, the read depth that would be satisfactory to obtain such genotypes (in our case at least 600 reads per amplicon) is really important to exclude sequencing errors [15]. 

Recently, there have been many publications pointing to errors that can occur during the NGS sequencing process [14,16]. It is important to recognize that long-term limitations of genotyping by sequencing can also occur because, similar to traditional SSCP/Sanger genotyping, approaches of NGS sequencing are still based on amplification of the target DNA sequence by PCR. The variety of artefacts that occur during the PCR step can far exceed the number of variants corresponding to true alleles, and the total number of sequence reads derived from artefacts can be higher than those from true alleles [33]. PCR may also be responsible for the allelic dropout caused by differences in amplification efficiency between alleles, especially in the SSCP/Sanger methodology [34]. Allelic dropouts and false alleles can cause important genotyping errors and thus significantly alter downstream analyses based on heterozygosity, allele diversity, and genotype composition, which has a direct implication in population genetics.

The stochastic variation of allele frequencies will inevitably worsen in NGS methodologies if the number of reads is reduced. Ion Torrent sequencing is currently one of the methods of choice for MHC genotyping but, for accurate genotyping, it is important to cover a sufficient number of reads per amplicon and to use strict approaches for the quality control of generated data and allele calling [12]. Using Illumina sequencing, Biedrzycka et al. [15] tested four genotyping approaches for characterizing MHC diversity in a passerine bird with the maximum number of co-amplifying alleles exceeding 40 per individual. The four different genotyping methods tested in their study were developed based on different principles: (i) set a threshold based on the observed frequencies of variants that could be explained as a sequencing error [35]; (ii) use two amplicon replicates to separate true alleles from artefacts [33]; (iii) set a threshold between true alleles and artefacts based on the drop in sequencing depth between them [11]; and (iv) use the error rate of a particular HTS platform to cluster true alleles with their potential artefacts [26]. Despite these differences, the authors found generally high agreement across genotyping methods and demonstrated that HTS enables reliable genotyping of highly complex MHC systems if sufficient coverage per amplicon is reached (in their case, at least 5000 reads per amplicon).

NGS technologies are likely to replace cloning and Sanger sequencing for MHC genotyping. However, traditional genotyping methods may remain an alternative that should be considered for genotyping a limited number of samples, as NGS is still expensive, and many research groups do not have direct access to these technologies. Our study has shown that the results of the Ion Torrent and traditional cloning/Sanger sequencing are comparable on a qualitative level, but with NGS, we were able to detect a higher number of “putative alleles”.

We must emphasize that this discrepancy between NGS and traditional methods is probably species-specific and depends on the complexity of the MHC II *DRB* system under investigation. The chamois MHC II *DRB* system is quite simple with only one functional copy of the gene, but the difference between the methods can be much bigger in more complex systems [15]. 

In our opinion, the higher probability of allele detection with NGS is based on the higher sequencing depth, which can compensate for the limitations of SSCP/Sanger sequencing, since the difficult amplification of some alleles i.e., differences in allele’s amplification efficiency, is a real limitation of this traditional method.

The correct assessment of an individual’s MHC constitution is the most fundamental prerequisite for understanding the functional significance of MHC allele diversity in evolutionary ecology, pathogen resistance, and conservation. The comparison of the methodological approaches is necessary to enable researchers to evaluate in detail and understand the data generated by NGS, which will improve confidence in the approach and in subsequent analyses and applications.

## 5. Conclusions

The major histocompatibility complex is polymorphic and polygenic in most species and therefore inherently difficult to genotype. We have shown that NGS technologies are better performing in sequencing highly variable regions such as the MHC compared to the SSCP/Sanger methodology, but strict pipelines for allele calling are required to distinguish true alleles from artefacts. Since SSCP/Sanger genotyping may underestimate the actual MHC variability, this method will most likely have very limited applicability. However, SSCP/Sanger analysis may remain an alternative for genotyping a limited number of samples and may be applicable to species with a less complex MHC system, such as chamois.

## Figures and Tables

**Table 1:** *DRB* exon 2 allele frequencies estimated with Single-Strand Conformation Polymorphism (SSCP)/Sanger sequencing and amplicon-based next-generation sequencing (NGS).

Allele	SSCP/Sanger		NGS	
	N^o^ Observations	Allele Frequency	N^o^ Observations	Allele Frequency
Ruru-DRB*01	20	0.357	18	0.321
Ruru-DRB*04	18	0.321	17	0.304
Ruru-DRB*09	1	0.018	1	0.018
Ruru-DRB*18	5	0.089	6	0.107
Ruru-DRB*41	0	0.000	1	0.018
Ruru-DRB*42	2	0.036	2	0.036
Ruru-DRB*43	6	0.107	4	0.071
Rupy-DRB1*04	4	0.071	7	0.125

## Supplementary Materials

The following are available online at https://www.mdpi.com/2076-2615/10/9/1694/s1, Figure S1: Variants ordered by descending per amplicon frequency after AmpliSAS clustering, Figure S2: Percentages of amplicon reads assigned to alleles before and after AmpliSAS clustering, Figure S3: Representation of amplicon depths from 28 individuals, and the number of reads of true sequences and artefacts within each amplicon, Table S1: AmpliSAS genotyping results of 28 chamois individuals, Table S2: Amplicon depths from 28 individuals, and the number of reads of true sequences and artefacts within each amplicon, Table S3: A summary of the DRB alleles found in each chamois individual using single-strand conformation polymorphism (SSCP)/Sanger sequencing and amplicon‐based next‐generation sequencing (NGS).
