# Supplementary material for: MHC Genotyping by SSCP and Amplicon-Based NGS Approach in Chamois
Source: Animals (Basel). 2020 Sep 18;10(9):1694. doi: 10.3390/ani10091694 (PMC7552744; doi:10.3390/ani10091694)
Supplement: Supplementary file 1 [file animals-10-01694-s001.zip › animals-912355-supplementary-final-update/animals-912355-Supplementary-figures-proofreading_Sep_18.docx]

Communication

MHC Genotyping by SSCP and Amplicon‐Based NGS Approach in Chamois

Sunčica Stipoljev, Elena Bužan, Barbora Rolečková, Laura Iacolina and Nikica Šprem

**Figure S1.** Variants ordered by descending per amplicon frequency (PAF) after sequencing errors have been added to them through the clustering step performed by AmpliSAS [1]. Lines represent amplicons of 28 individuals. Horizontal line represents 14% PAF threshold for the filtering step [1].

**Figure S2.** Percentages of amplicon reads assigned to alleles before and after AmpliSAS clustering. On average, 54% of amplicon depths were assigned to alleles, and after clustering this proportion increased to 83%.

**Figure S3.** Representation of amplicon depths of 28 individuals, and the number of reads of true sequences and artefacts within each amplicon. True—sequences that match a real allele i.e. true variant; the following are sequencing errors: X—1bp substitutions, I—insertions, D—deletions, H—homopolymer indels; XIDH—sequence with any combination of at least two sequencing errors; leftovers - low frequency variants, chimeras or sequences containing many errors which could not be classified into the major clusters. All sequencing errors, except leftovers, were clustered with the true variant from which they were derived.

Reference

1. Sebastian, A.; Herdegen, M.; Migalska, M.; Radwan, J. Amplisas: A web server for multilocus genotyping using next-generation amplicon sequencing data. *Mol. Ecol. Resour.* **2016**, *16*, 498–510, doi:10.1111/1755-0998.12453.

| 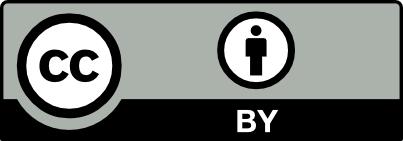 | © 2020 by the authors. Licensee MDPI, Basel, Switzerland. This article is an open access article distributed under the terms and conditions of the Creative Commons Attribution (CC BY) license (http://creativecommons.org/licenses/by/4.0/). |
| --- | --- |
